# Supplementary material for: Optical gap and fundamental gap of oligoynes and carbyne
Source: Nat Commun. 2020 Sep 23;11:4797. doi: 10.1038/s41467-020-18496-4 (PMC7511338; doi:10.1038/s41467-020-18496-4)
Supplement: Supplementary file 1 — Supplementary Information [file 41467_2020_18496_MOESM1_ESM.pdf]

Supplementary Information

Optical Gap and Fundamental Gap of Oligoynes and Carbyne

Zirzlmeier et al.

Table of Contents

1.	Supplementary Figures 1–19	S3
2.	Supplementary Tables 1–4	S14
3.	Supplementary Method 1	S16
4.	Supplementary Method 2	S19
5.	Supplementary Method 3	S23
6.	Supplementary References	S27

1. Supplementary Figures 1–19

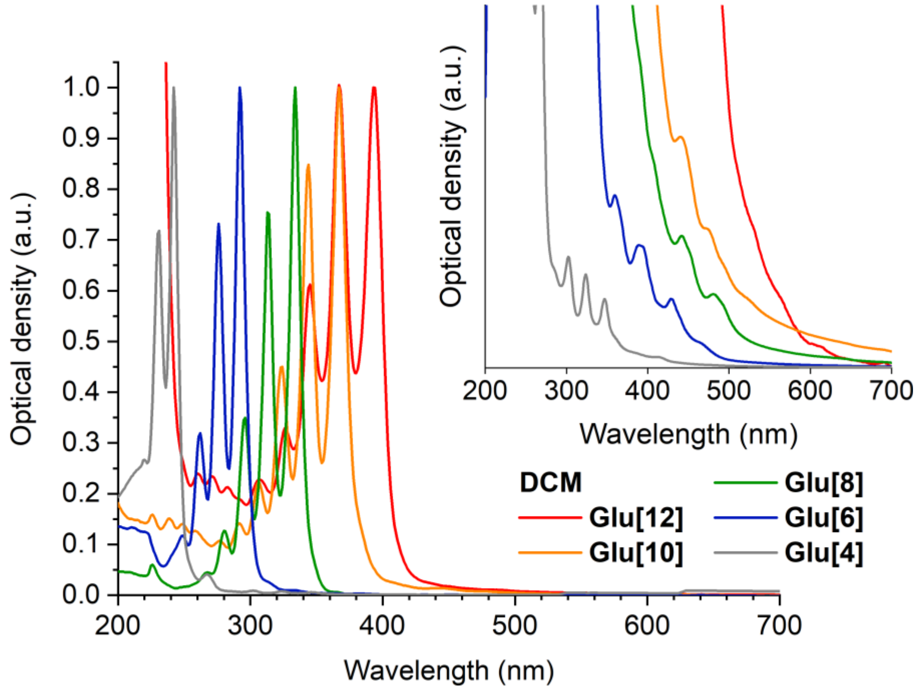

Supplementary Figure 1. Normalized absorption spectra of the **Glu[*n*]** series recorded in dichloromethane (DCM). The insets show the additional bands with weak intensity (λ_{weak}) at wavelengths higher than the longest wavelength absorption maximum (λ_{main}). Source data are provided as a Source Data file.

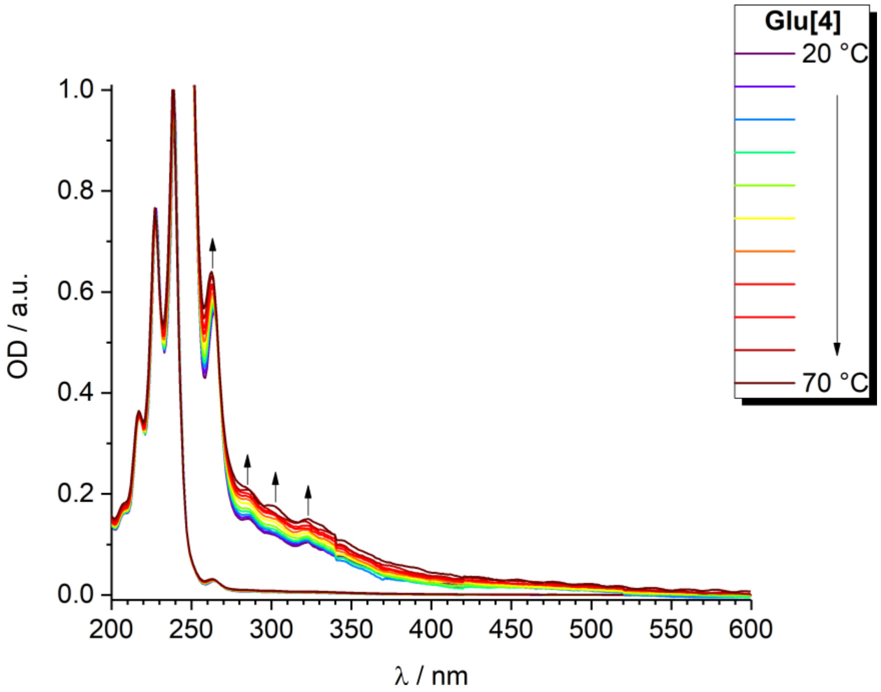

Supplementary Figure 2. Temperature dependent steady-state absorption spectra of **Glu[4]** measured in acetonitrile (the signals (λ_{weak}) of have been magnified). The arrows indicate the increasing absorption intensities with increasing temperature. Source data are provided as a Source Data file.

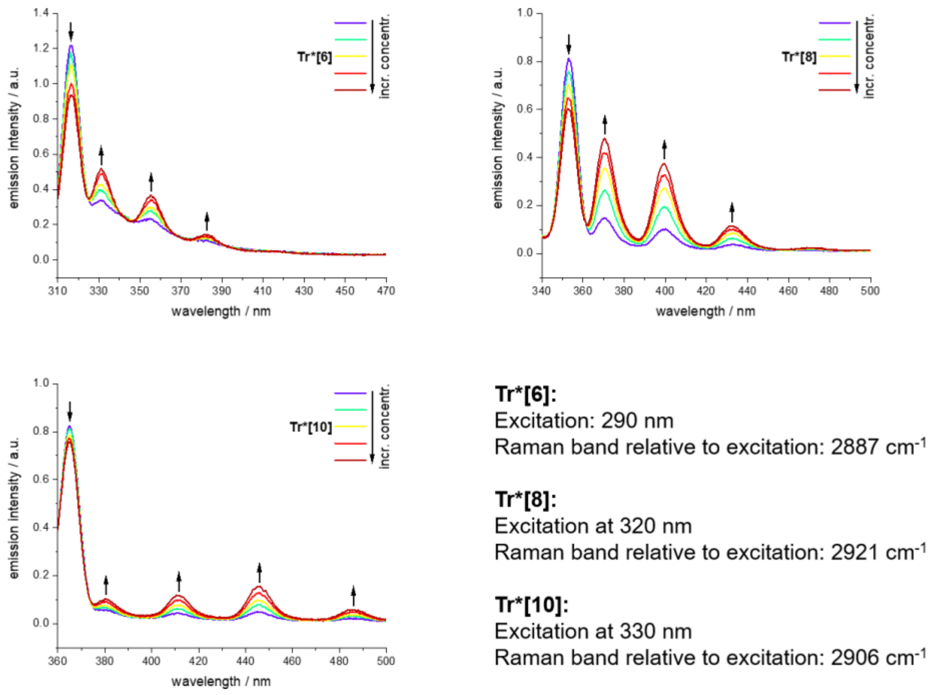

Supplementary Figure 3. Concentration dependent steady-state fluorescence spectra of **Tr*[*n*]** with n = 6-10 recorded in hexane. The solvent related Raman features, which are 2800-3000 cm-1 red-shifted relative to the excitation wavelength are marked with an arrow pointing downward whereas the upward arrows indicate bands that show an increasing emission intensity with increasing concentrations. See figure legend for further details. Source data are provided as a Source Data file.

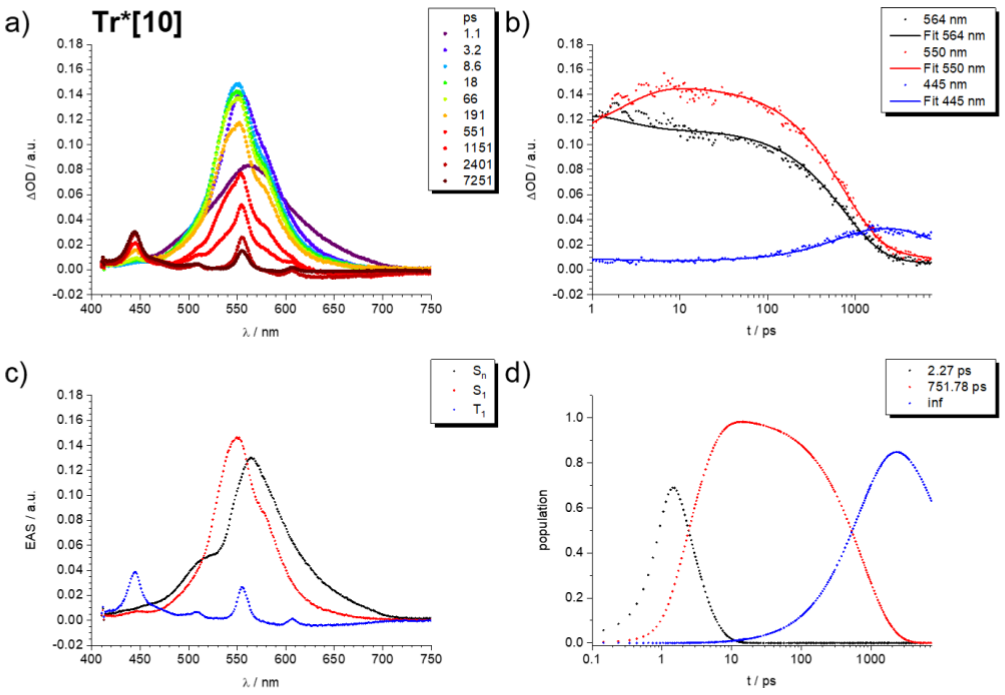

Supplementary Figure 4. a) Differential absorption spectra (visible) obtained upon fs pump-probe experiments (@ 258 nm) of **Tr*[10]** in hexane with several time delays between 1.1 and 7251 ps at room temperature. **b)** Time absorption profiles of the spectra at 564 (black), 550 (red), and 445 nm (blue) monitoring the excited state dynamics. **c)** Evolution associated spectra (EAS) as obtained by global analysis (GA). **d)** Evolution of the population of the respective species as obtained by GA. Source data are provided as a Source Data file.

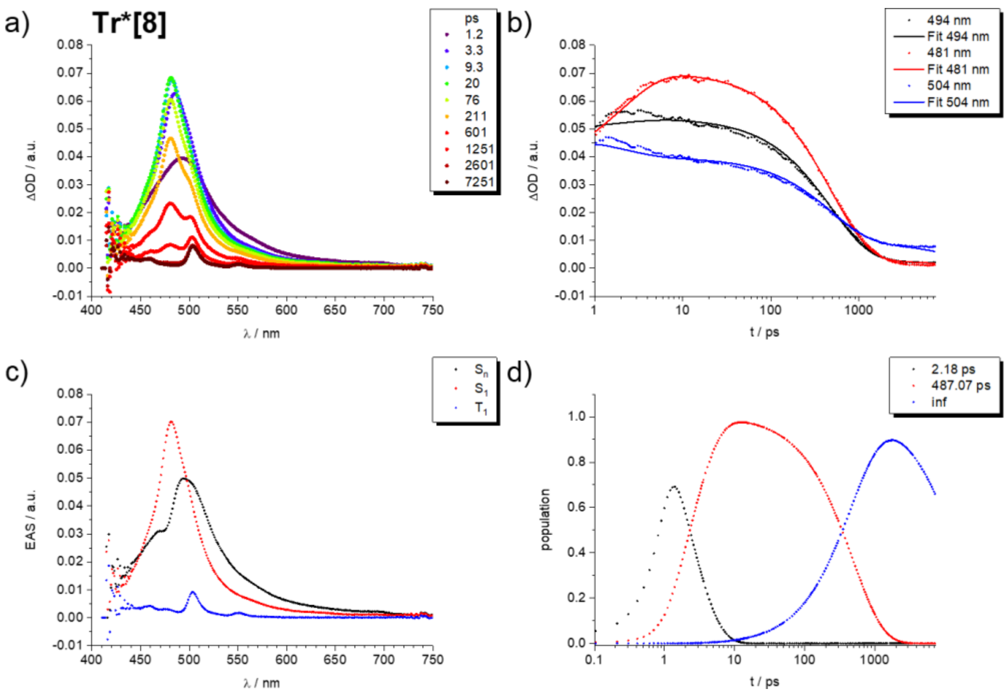

Supplementary Figure 5. a) Differential absorption spectra (visible region) obtained upon fs pump-probe experiments (@ 258 nm) of **Tr*[8]** in hexane with several time delays between 1.2 and 7251 ps at room temperature. **b)** Time absorption profiles of the spectra at 494 (black), 481 (red), and 504 nm (blue) monitoring the excited state dynamics. **c)** Evolution associated spectra (EAS) as obtained by global analysis (GA). **d)** Evolution of the population of the respective species as obtained by GA. Source data are provided as a Source Data file.

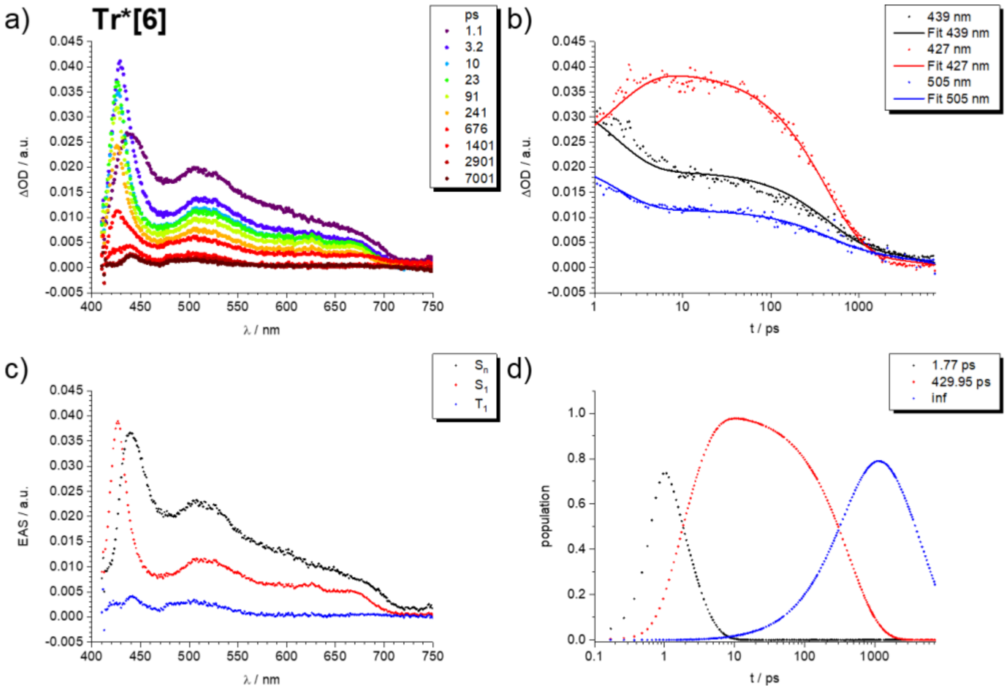

Supplementary Figure 6. a) Differential absorption spectra (visible region) obtained upon fs pump-probe experiments (@ 258 nm) of **Tr*[6]** in hexane with several time delays between 1.1 and 7001 ps at room temperature. **b)** Time absorption profiles of the spectra at 439 (black), 427 (red), and 505 nm (blue) monitoring the excited state dynamics. **c)** Evolution associated spectra (EAS) as obtained by global analysis (GA). **d)** Evolution of the population of the respective species as obtained by GA. Source data are provided as a Source Data file.

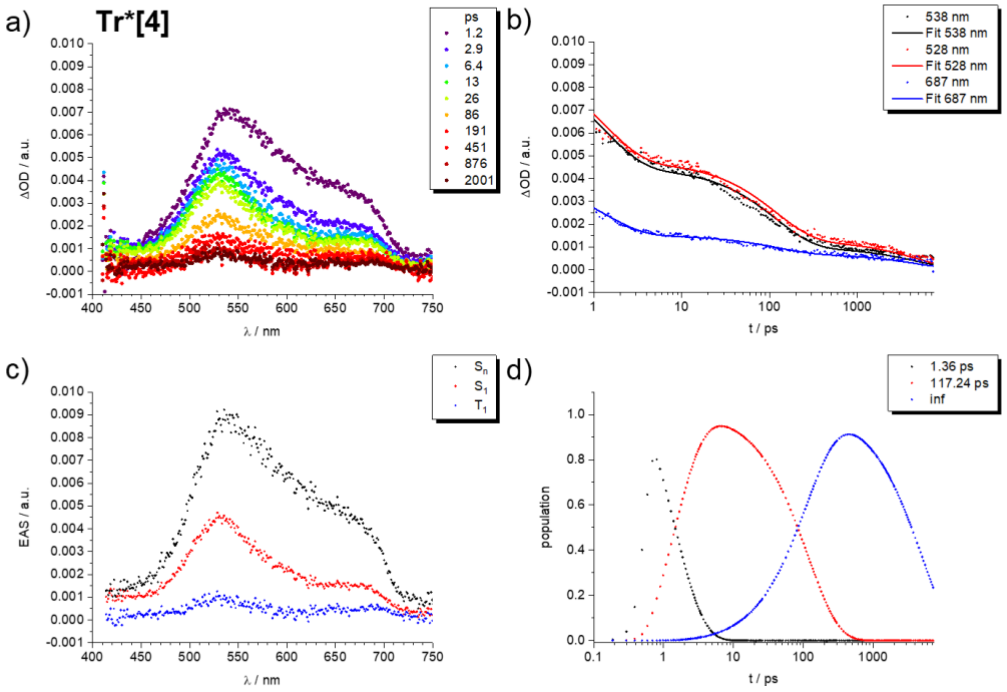

Supplementary Figure 7. a) Differential absorption spectra (visible region) obtained upon fs pump-probe experiments (@ 258 nm) of **Tr*[4]** in hexane with several time delays between 1.2 and 2001 ps at room temperature. **b)** Time absorption profiles of the spectra at 538 (black), 528 (red), and 687 nm (blue) monitoring the excited state dynamics. **c)** Evolution associated spectra (EAS) as obtained by global analysis (GA). **d)** Evolution of the population of the respective species as obtained by GA. Source data are provided as a Source Data file.

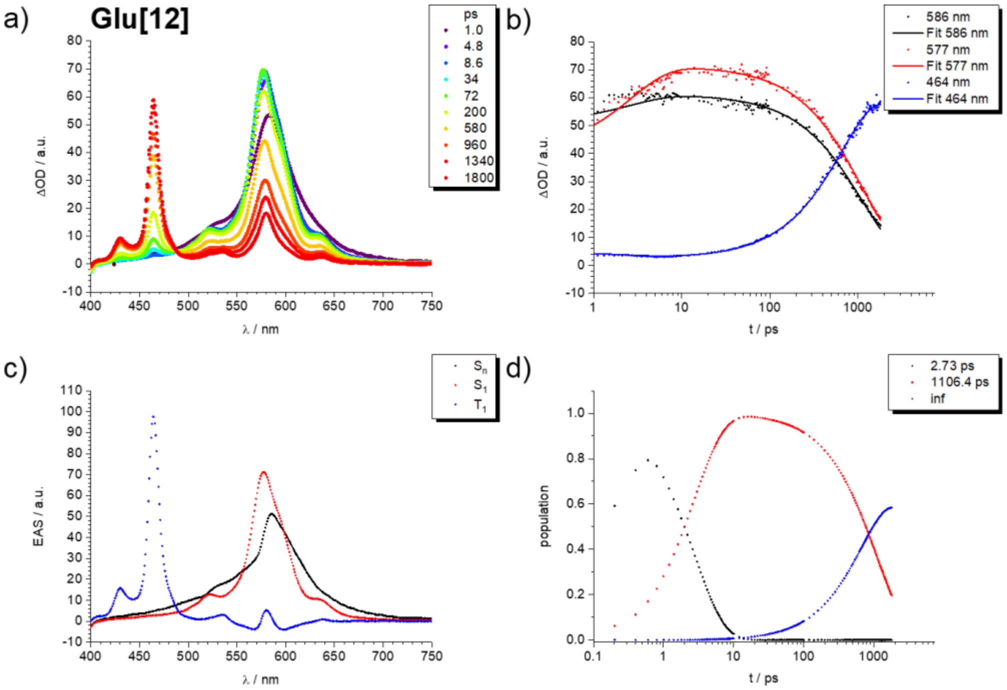

Supplementary Figure 8. a) Differential absorption spectra (visible) obtained upon fs pump-probe experiments (@ 390 nm) of **Glu[12]** in dichloromethane with several time delays between 1.0 and 1800 ps at room temperature. **b)** Time absorption profiles of the spectra at 586 (black), 577 (red), and 464 nm (blue) monitoring the excited state dynamics. **c)** Evolution associated spectra (EAS) as obtained by global analysis (GA). **d)** Evolution of the population of the respective species as obtained by GA. Source data are provided as a Source Data file.

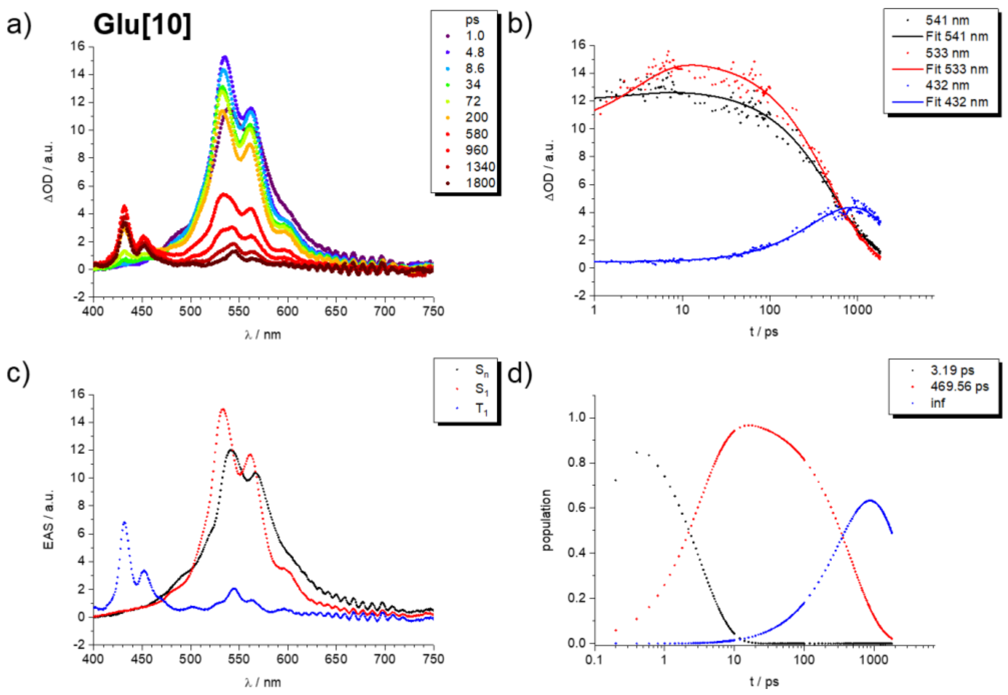

Supplementary Figure 9. a) Differential absorption spectra (visible) obtained upon fs pump-probe experiments (@ 390 nm) of **Glu[10]** in dichloromethane with several time delays between 1.0 and 1800 ps at room temperature. **b)** Time absorption profiles of the spectra at 541 (black), 533 (red), and 432 nm (blue) monitoring the excited state dynamics. **c)** Evolution associated spectra (EAS) as obtained by global analysis (GA). **d)** Evolution of the population of the respective species as obtained by GA. Source data are provided as a Source Data file.

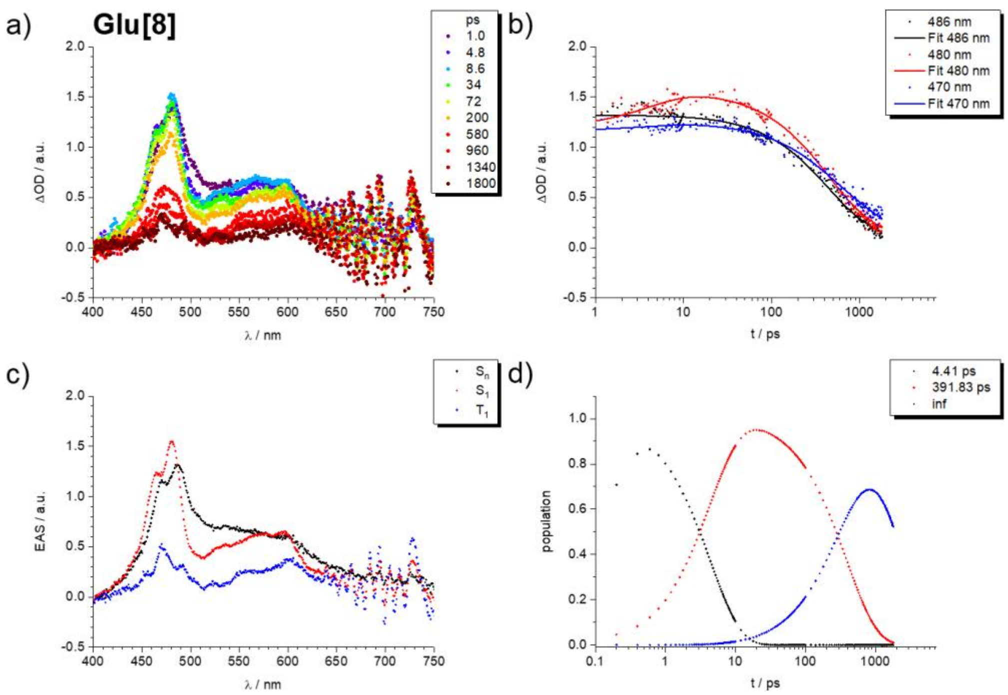

Supplementary Figure 10. a) Differential absorption spectra (visible) obtained upon fs pump-probe experiments (@ 390 nm) of **Glu[8]** in dichloromethane with several time delays between 1.0 and 1800 ps at room temperature. **b)** Time absorption profiles of the spectra at 486 (black), 480 (red), and 470 nm (blue) monitoring the excited state dynamics. **c)** Evolution associated spectra (EAS) as obtained by global analysis (GA). **d)** Evolution of the population of the respective species as obtained by GA. Source data are provided as a Source Data file.

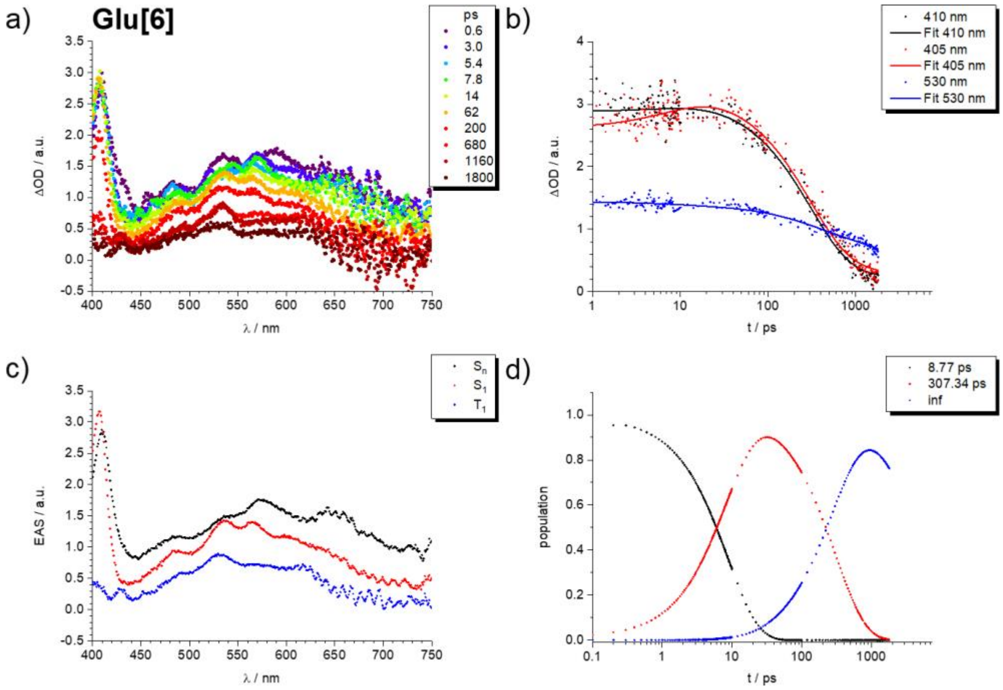

Supplementary Figure 11. a) Differential absorption spectra (visible) obtained upon fs pump-probe experiments (@ 390 nm) of **Glu[6]** in dichloromethane with several time delays between 0.6 and 1800 ps at room temperature. **b)** Time absorption profiles of the spectra at 410 (black), 405 (red), and 530 nm (blue) monitoring the excited state dynamics. **c)** Evolution associated spectra (EAS) as obtained by global analysis (GA). **d)** Evolution of the population of the respective species as obtained by GA. Source data are provided as a Source Data file.

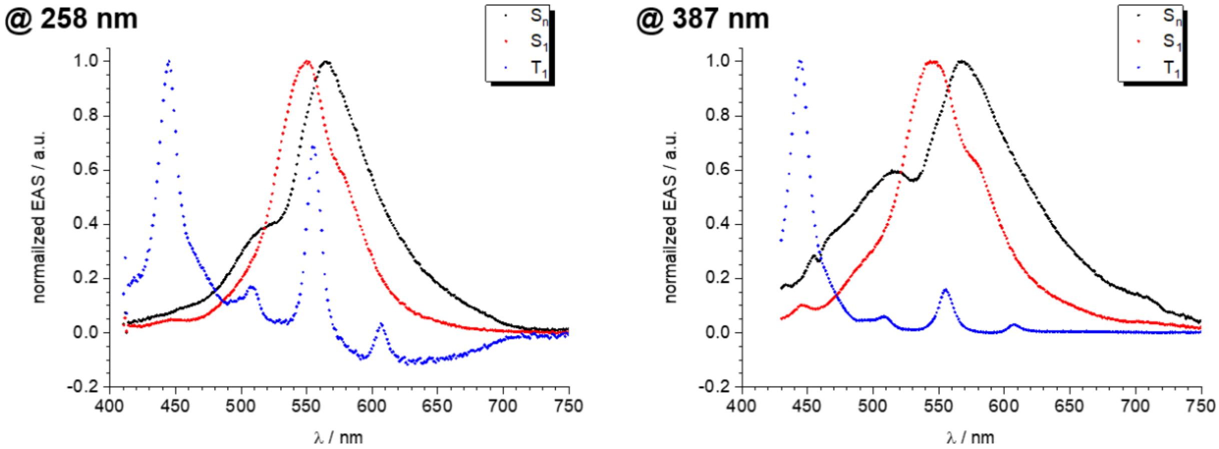

Supplementary Figure 12. Normalized evolution associated spectra (EAS) as obtained by global analysis (GA) of the differential absorption data (visible detection range) obtained upon fs pump-probe experiments of **Tr*[10]** in hexane upon excitation @ 258 nm (left) and @ 387 nm (right). Source data are provided as a Source Data file.

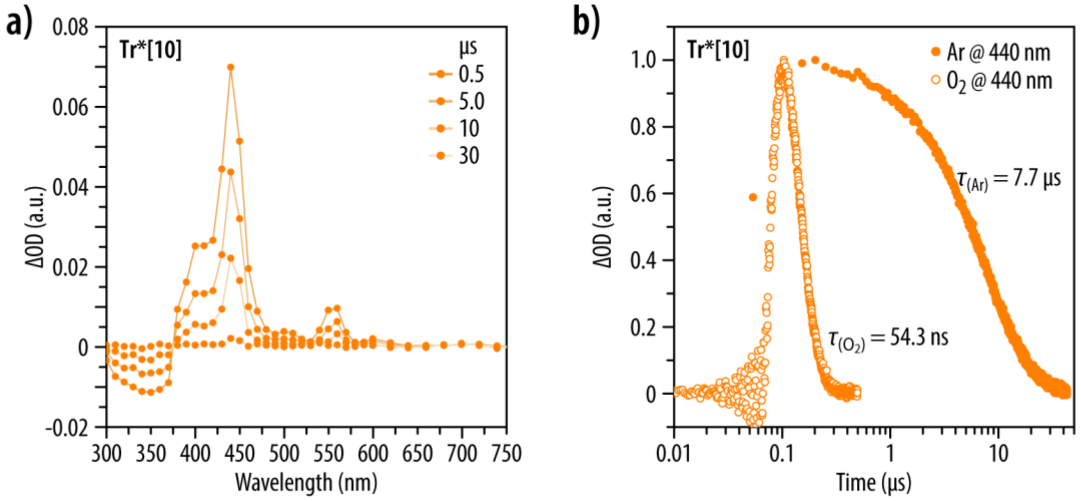

Supplementary Figure 13. a) Differential absorption spectra (visible region) obtained upon nanosecond pump-probe experiments (upon excitation at 266 nm) of **Tr*[10]** in hexane with different time delays between 0.5 and 30 μs at room temperature. **b)** Time absorption profiles of the spectra at 440 nm monitoring the excited state dynamics in argon-saturated (closed circles) and oxygen-saturated (open circles) solution. Source data are provided as a Source Data file.

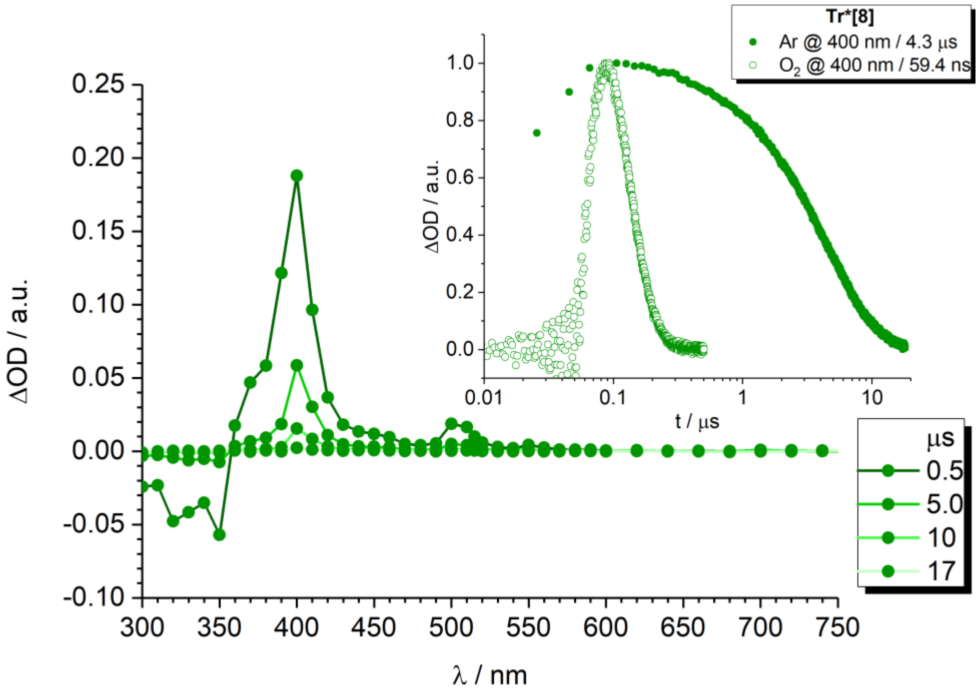

Supplementary Figure 14. Differential absorption spectra (visible region) obtained upon ns pump-probe experiments (@ 266 nm) of **Tr*[8]** in hexane with several time delays between 0.5 and 17 μs at room temperature. **Inset:** Time absorption profiles of the spectra at 400 nm monitoring the excited state dynamics in argon-saturated (closed circles) and oxygen-saturated (open circles) solution. Source data are provided as a Source Data file.

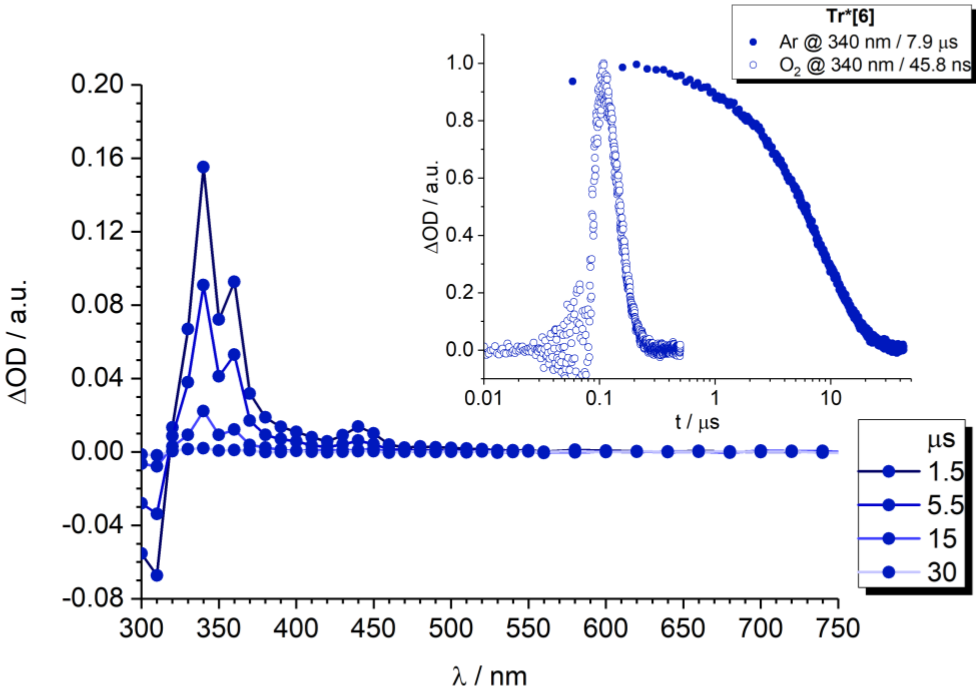

Supplementary Figure 15. Differential absorption spectra (visible region) obtained upon ns pump-probe experiments (@ 266 nm) of **Tr*[6]** in hexane with several time delays between 1.5 and 30 μs at room temperature. **Inset:** Time absorption profiles of the spectra at 340 nm monitoring the excited state dynamics in argon-saturated (closed circles) and oxygen-saturated (open circles) solution. Source data are provided as a Source Data file.

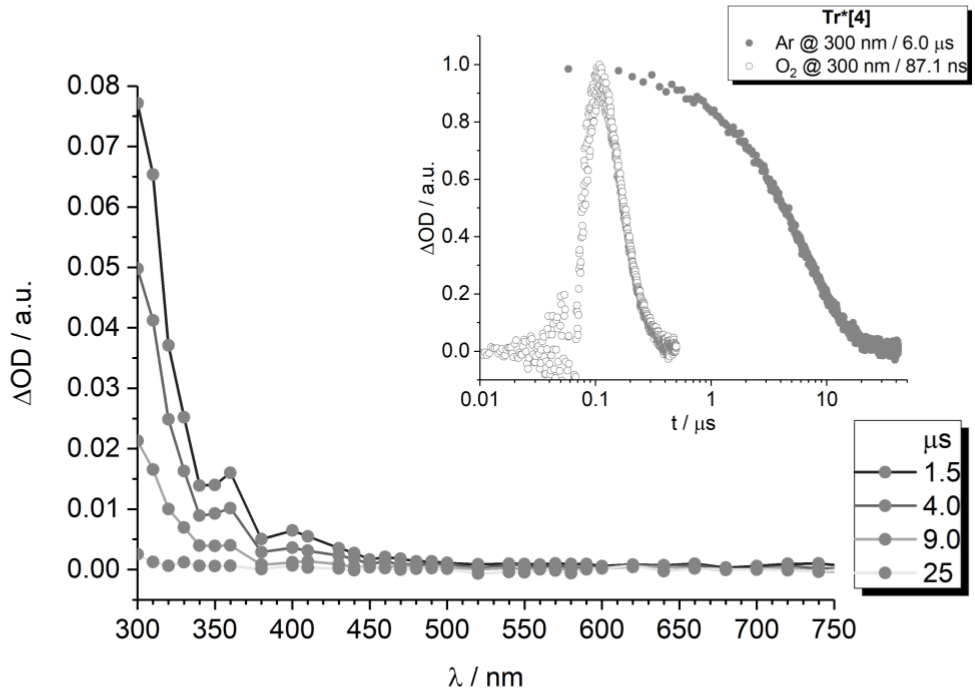

Supplementary Figure 16. Differential absorption spectra (visible region) obtained upon ns pump-probe experiments (@ 266 nm) of **Tr*[4]** in hexane with several time delays between 1.5 and 25 μs at room temperature. **Inset:** Time absorption profiles of the spectra at 300 nm monitoring the excited state dynamics in argon-saturated (closed circles) and oxygen-saturated (open circles) solution. Source data are provided as a Source Data file.

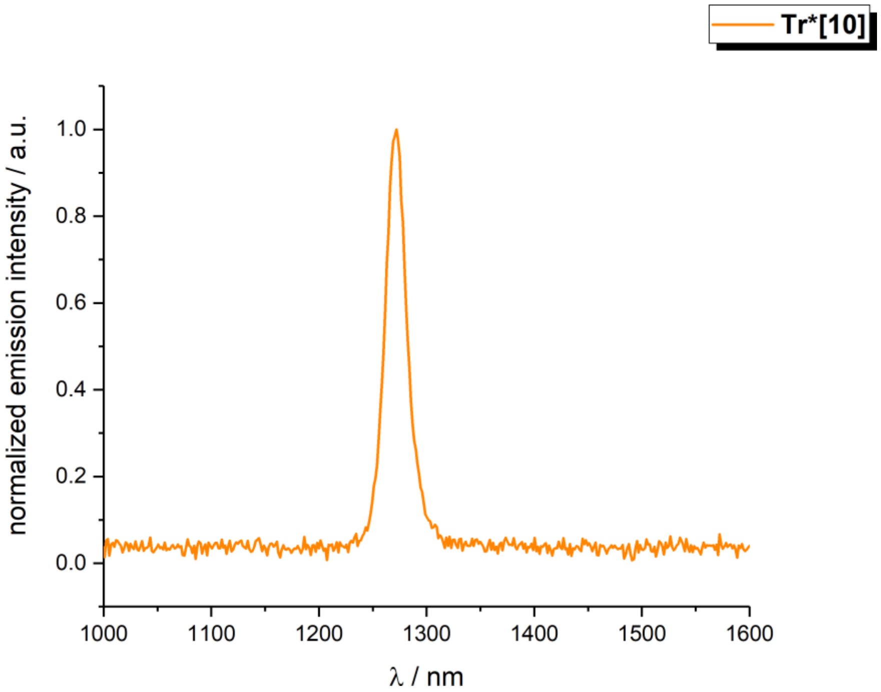

Supplementary Figure 17. Singlet oxygen phosphorescence spectrum of **Tr*[10]** measured in oxygen saturated hexane upon excitation at 315 nm (OD = 0.15). Source data are provided as a Source Data file.

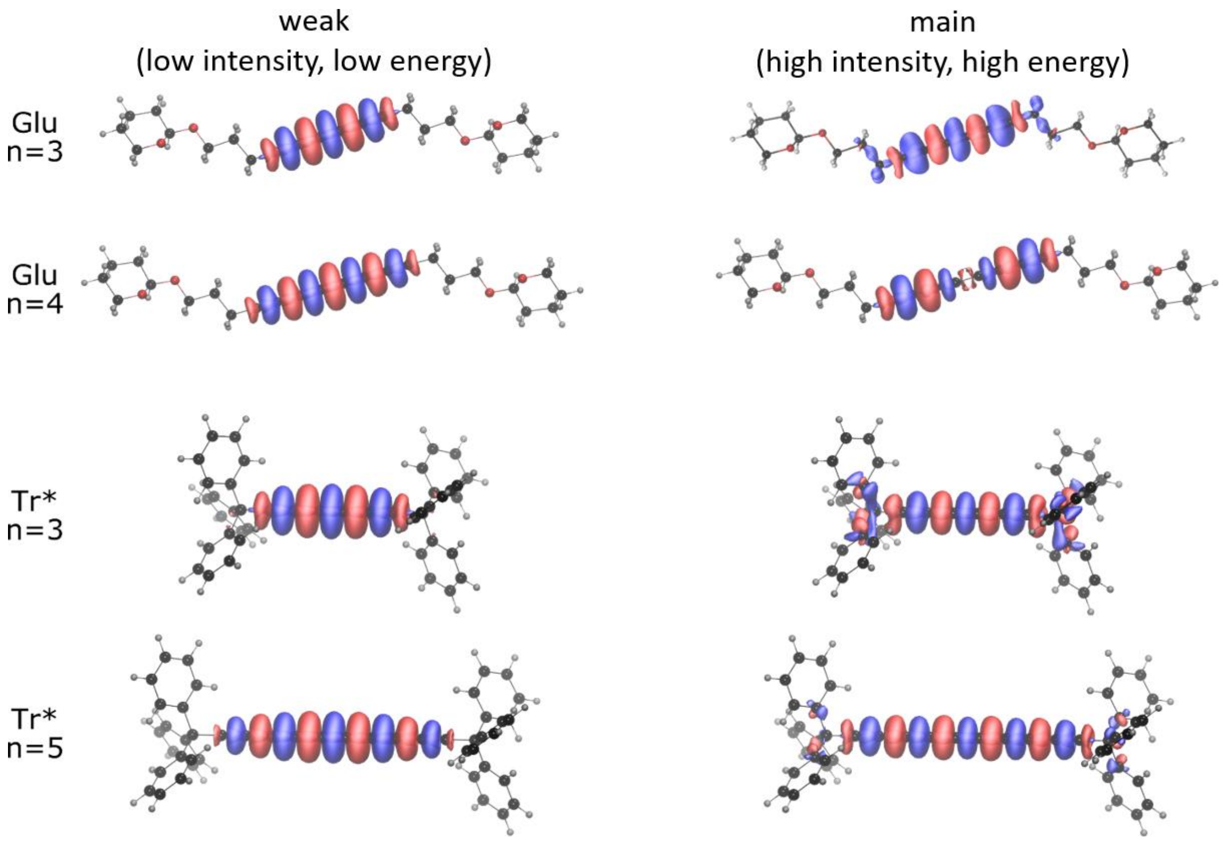

Supplementary Figure 18. Depiction of the electron density differences between the excited and ground states (ES-GS) for the high and low transition of **Glu[*n* = 3, 4]** and **Tr*[*n*=3, 5]** computed at the CAM-B3LYP/6-31+G(d) level on CAM-B3LYP/6-31G(d) geometries ($\Delta\rho(\mathbf{r})$ isocontours: -0.001(blue) and +0.001(red)).

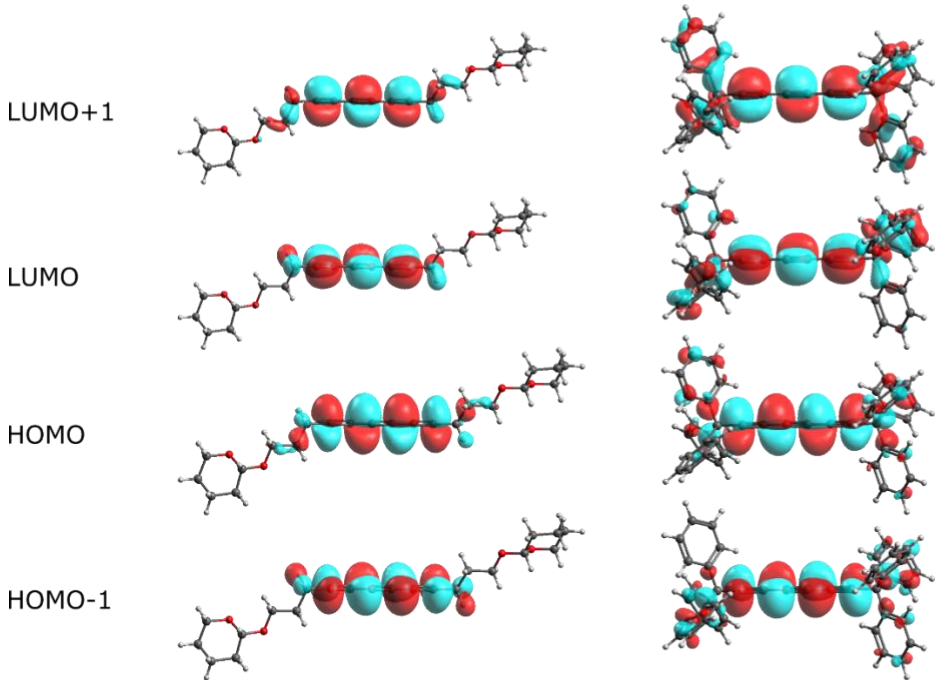

Supplementary Figure 19. Orbitals participating to the main optical transitions of **Glu[3]** (left) and **Tr*[3]** (right, isovalues = ±0.02) computed at the CAM-B3LYP/6-31+G(d) level on CAM-B3LYP/6-31G(d) geometries. While S_2 and S_{10} share the same orbital excitations in **Tr*[3]**, the sign of configuration interaction differs, which explains the difference in oscillator strength. For S_2, the transition dipole moments largely cancel, while for S_{10} they add up constructively, leading to a bright S_{10} state (see Supplementary Tables 3–4, and Supplementary Figure 18 for detailed orbital contributions and representations). The same explanation holds for the whole **Tr*[*n*]** as well as **Glu[*n*]** oligoyne series considering the seemingly identical bright and dark excitations.

2. Supplementary Tables 1–4

Supplementary Table 1. Experimental data determined by absorption spectroscopy for **Glu[*n*]** with *n* = 4–12 (acetonitrile) and **Tr*[*n*]** with *n* = 4–10 (hexane).[1] Source data are provided in the Source Data file.

Compound	**Tr*[*n*]**		**Glu[*n*]**		
	$S_0 \rightarrow S_n$ (λ_{main}) [nm]	ε $S_0 \rightarrow S_n$ [M^{-1} cm^{-1}]	$S_0 \rightarrow S_n$ (λ_{main}) [nm]	ΔE ($S_0 \rightarrow S_n$) [cm^{-1}]	$S_0 \rightarrow S_{2/3}$ (λ_{weak}) [nm]
n = 4	268	149000	239	2019	345
n = 6	310	412000	288	2043	427
n = 8	347	541000	329	1967	479
n = 10	376	595000	361	1885	513
n = 12	–	–	386	1871	579

Supplementary Table 2. Experimental data determined by fluorescence spectroscopy for the **Tr*[*n*]** series of oligoyne derivatives with *n* = 6, 8, 10 (hexane; n.d. = not determined). Source data are provided in the Source Data file.

Compound	λ_{em} [nm]	Stokes shift [cm^{-1}][a]	Φ_F [10^{-4}]
**Tr*[6]**	331	n.d.	2.0
**Tr*[8]**	371	n.d.	3.9
**Tr*[10]**	380	280	1.8

[a] An exact value for the Stokes shift [cm^{-1}] can only be given for **Tr*[10]** – see also Supplementary Figure 3.

Supplementary Table 3. Excitation energies (*E*), oscillator strengths (*f*), orbital contributions and CI coefficients for the main optical transitions of **Glu[3]**, computed at the CAM-B3LYP/6-31+G(d) level on CAM-B3LYP/6-31G(d) geometries.

**Transition**	*E* (eV)	*f*	Excitation	Contrib. (%)	CI coeff.
**S_2**	4.08	0.0017	HOMO–1 → LUMO<br>HOMO → LUMO+1	50<br>48	0.5<br>0.49
**S_3**	4.09	0.0003	HOMO–1 → LUMO+1<br>HOMO → LUMO	72<br>25	0.6<br>–0.35
**S_5**	6.18	4.0868	HOMO–1 → LUMO<br>HOMO → LUMO+1	42<br>42	–0.46<br>0.46

Supplementary Table 4. Excitation energies (*E*), oscillator strengths (f), orbital contributions and CI coefficients for the main optical transitions of **Tr*[3]**, computed at the CAM-B3LYP/6-31+G(d) level on CAM-B3LYP/6-31G(d) geometries.

**Transition**	*E* [eV]	f	Excitation	Contrib. (%)	CI coeff.
**S_2**	4.01	0.0067	HOMO–1 → LUMO<br>HOMO → LUMO+1	42<br>42	0.46<br>0.46
**S_3**	4.01	0.0067	HOMO–1 → LUMO+1<br>HOMO → LUMO	42<br>42	0.46<br>–0.46
**S_{10}**	5.58	3.44	HOMO–1 → LUMO<br>HOMO → LUMO+1	37<br>37	–0.43<br>0.43

3. Supplementary Method 1

Glu[n] series – Steady-state absorption. Steady-state UV-Vis spectra of solutions were recorded with a scan speed of 400 nm per minute on a JASCO V670 spectrometer using 1 cm quartz cuvettes from Hellma. For measurements at elevated temperatures, the spectrometer was equipped with a JASCO ETCR-762 temperature controller connected to a JASCO MCB-100 mini circulation bath.

Glu[n] series – Femtosecond transient absorption. Transient absorption spectra of solutions were recorded using femtosecond pulsed laser pump-probe spectroscopy. The solutions were measured in a 1 mm cell and constantly bubbled with argon gas to provide stirring and prevent degradation due to oxygen. The probe beam consisted of a white light continuum (350 nm to 950 nm), generated by passing a part of the 780 nm amplified 1 kHz Ti:sapphire output (Clark-MXR, CPA-2001) through a c-cut 3 mm thick sapphire plate. The remaining intensity of the fundamental has been removed by a 750 nm low pass filter. The excitations pulses, at 390 nm, have been generated by frequency doubling of the fundamental of the laser. The probe intensity was always less than the pump intensity and the spot size much smaller to allow for a homogeneous excitation over the probed area. The pump pulses were delayed with respect to the probe pulses using a computerized translation stage. The probe pulses were split before the sample by means of a beam splitter into a signal (transmitted through the sample) a reference beam. The probe and reference beam were detected separately using a pair of 163 m spectrographs (Andor Technology, SR163) equipped with 512 x 58 pixel back-thinned CCDs (Hamamatsu S07030-0906) and assembled by Entwicklungsbüro Stresing, Berlin. The pump beam was chopped at half the amplifier frequency to improve the sensitivity of the set-up. The transmitted intensity of the probe beam was recorded shot by shot and corrected for laser intensity fluctuations using the reference beam. The transient spectra were averaged at each delay until the desired signal-to-noise ratio was reached (3000 times). To avoid polarization effects the relative polarization of the probe and pump pulses was set to the magic angle of 54.7°. All spectra were corrected for the chirp of the white-light probe.

Tr*[n] series – Steady-state absorption and emission. Steady-state UV-Vis absorption spectra of solutions were acquired at room temperature using a Lambda 2 spectrometer (Perkin Elmer); 190 to 1100 nm, double-beam-instrument. Steady-state fluorescence spectra of solutions were measured with a FluoroMax3 spectrometer (Horiba) in the visible detection range (rt) and a FluoroLog3 spectrometer (Horiba) equipped with an IGA Symphony (512 x 1 x 1 µm) detector in the near-infrared detection range. Fluorescence quantum yields Φ_F were determined by means of gradient analyses using [1,4-bis-(5-phenyloxazol-2-yl)benzene] in cyclohexane (POPOP, Φ_F = 0.975)[2] as reference compound.

Tr*[n] series – Femtosecond transient absorption. Femtosecond transient absorption (fsTA) spectra (rt) of solutions were recorded using femtosecond pulsed laser pump-probe spectroscopy. The solutions were measured in a 2 mm cell and bubbled with argon gas prior to the measurement to prevent degradation and quenching by oxygen. The probe beam consisted of a white light continuum (420 – 1600 nm), generated by passing a part of the 775 nm amplified 1.05 kHz Ti:sapphire output (Clark-MXR, CPA-2101, 150 fs pulse width) through a c-cut 2 mm thick sapphire plate. The remaining intensity of the fundamental has been removed by a 750 nm low pass filter and an 850 nm long pass filter for the visible and the near-infrared region, respectively. The excitation pulses, at 258 nm, were generated by frequency tripling of the fundamental of the laser. Transient absorption spectra were recorded using transient absorption pump / probe detection systems (Helios and Eos, Ultrafast Systems). The probe intensity was always less than the pump intensity and the spot size much smaller to allow for a homogeneous excitation over the probed area. The probe pulses were delayed with respect to the pump pulses using a computerized translation stage. The probe pulses were split before the sample by means of a beam splitter into a signal (transmitted through the sample) and a reference beam. The probe and reference beam were detected separately. The transmitted intensity of the probe beam was recorded shot by shot and corrected for laser intensity fluctuations using the reference beam. The transient spectra were averaged at each delay until the desired signal-to-noise ratio was reached (1500 times). To avoid polarization effects the

relative polarization of the probe and pump pulses was set to magic angle. All spectra were corrected for the chirp of the white-light probe.

Tr*[n] series – Nanosecond transient absorption. Nanosecond transient absorption (nsTA) experiments (rt) were carried out with a non-commercial setup built at the Friedrich-Alexander University Erlangen-Nürnberg. The solutions were measured in a 5 mm cell and bubbled with argon gas prior to the measurement to prevent degradation and quenching by oxygen. As white light source (300 – 1200 nm), a pulsed 450 W XBO lamp (Osram) was used. The 266 nm excitation was generated via fourth harmonic generation (FHG) of the 1064 nm laser output of a Nd:YAG laser system (Brilliand-Quantel, output 1064 nm, 5 ns pulse width). Signal detection was performed with a R928 photomultiplier tube (Hamamatsu Photonics) for the UV-Vis and an InGaAs photodiode (Nano5-Coherent) for the near-infrared. The signals were digitalized using a 1 GHz digital WavePro7100 oscilloscope (LeCroy). All measurements were also carried out under oxygen atmosphere.

Tr*[n] series – Analyses of the femtosecond transient absorption data. For the femtosecond transient absorption measurements, global analysis and fitting of the transient absorption data were performed with the help of the open-source software package Glotaran.[3] The latter is a Java-based graphical user interface to the R package TIMP.[4,5] TIMP was developed for global and target analysis of time-resolved spectroscopy data. The dispersion of the instrument response function (IRF) was modeled and taken into account during the fitting procedure. Nanosecond transient absorption data were fitted using multiwavelengths analyses.

4. Supplementary Method 2

Instrumentation for chemical characterization. NMR Spectroscopy was carried out at 297.2 K on a Bruker Avance III 400 spectrometer at frequencies of 400.13 MHz for ^{1}H nuclei and 100.62 MHz for ^{13}C nuclei. Spectra were calibrated to the residual solvent peak of CD_2Cl_2 (5.32 ppm ^{1}H NMR; 54.00 ppm ^{13}C NMR) or $CDCl_3$ (7.26 ppm ^{1}H NMR; 77.16 ppm ^{13}C NMR). High resolution mass spectra were recorded as service measurements at the mass spectrometry service of the Institute of Chemical Sciences and Engineering at EPFL on a Thermo Scientific LTQ FT-ICR MS equipped with a Thermo Scientific Ion Max APCI/APPI source for atmospheric pressure photoionization (APPI).

General Synthetic Procedures. Unless otherwise noted, all reactions were carried out in dried Schlenk glassware in an inert argon atmosphere. Chromatography solvents were purchased as reagent grade and distilled once prior to use. For reactions, acetonitrile, dichloromethane, and tetrahydrofuran were purchased as HPLC grade and dried using a solvent purification system from Innovative Technologies. All reagents were commercially obtained and used without further purification. 2,6-Lutidine (99%) and copper(I) bromide (98%) were purchased from Acros Organics, cesium fluoride (99%) and silver(I) fluoride (99%) were purchased from Fluorochem, and copper(II) acetate monohydrate (99%) was purchased from Carl Roth. The asymmetric glycosylated oligoynes from the diyne to the hexayne as well as **Glu[2]**, **Glu[6]**, and series of oligoynes **Tr*[*n*]** were synthesized according to previously published procedures.[1,6] Derivatives higher than the symmetric hexayne **Glu[6]** were not isolated in the dried state, but kept in solution at all times. For analytical purposes, $CDCl_3$ (10 mL) was added, and the solution was concentrated *in vacuo*. **Caution:** The stability of oligoyne derivatives is strongly dependent on the end groups; although no explosions were encountered during the work described below, great care should be taken when carrying out similar reactions.

TLC analyses were performed on TLC plates from Merck (Silica gel 60 F_{254}). UV-light (254 nm) or anisaldehyde staining was used for detection. Column chromatography was conducted on Geduran silica gel Si 60 from Merck (40-60 µm).

Synthesis of tetradeca-4,6,8,10-tetrayne-1,14-diyl bis(2,3,4,6-tetra-*O*-acetyl-β-D-gluco-pyranoside) Glu[4]. In a flask shielded from light with aluminum foil, 7'-trimethylsilylhepta-4',6'-diynyl 2,3,4,6-tetra-*O*-acetyl-β-D-glucopyranoside (320.0 mg, 0.63 mmol) was dissolved in THF (100 mL) and water (20 mL). Cesium fluoride (123.8 mg, 0.82 mmol) was added, and the resulting mixture was stirred for 4 h. The mixture was diluted with ethyl acetate (200 mL), washed three times with saturated aqueous NH_4Cl solution, and once with saturated aqueous NaCl solution. The organic phase was dried over Na_2SO_4, concentrated *in vacuo* to a volume of ca. 10 mL, and purified by column chromatography (silica gel, EtOAc/heptane 2:1, R_f: 0.54). The combined fractions of the hepta-4',6'-diynyl 2,3,4,6-tetra-*O*-acetyl-β-D-glucopyranoside were concentrated *in vacuo* to a volume of ca. 20 mL, DCM (60 mL), $Cu(OAc)_2 \cdot H_2O$ (640.0 mg, 3.2 mmol), and 2,6-dimethylpyridine (2.2 mL, 18.9 mmol) were added, and the resulting mixture was stirred for 48 h. To this mixture 1 M HCl (100 mL) was added and the aqueous layer was extracted with DCM. The combined organic phases were washed with saturated NaCl solution, dried over Na_2SO_4, and concentrated *in vacuo*. Column chromatography (silica gel; EtOAc/*n*-pentane 4:1) yielded **Glu[4]** (179 mg, 65%) as a yellow solid.

TLC (EtOAc:*n*-pentane 80:20 v/v): R_f = 0.52; ^{1}H NMR (400 MHz, CD_2Cl_2) δ 5.19 (t, *J* = 9.6 Hz, 2H), 5.03 (t, *J* = 9.7 Hz, 2H), 4.91 (dd, *J* = 9.7, 8.0 Hz, 2H), 4.51 (d, *J* = 8.0 Hz, 2H), 4.24 (dd, *J* = 12.3, 4.9 Hz, 2H), 4.10 (dd, *J* = 12.3, 2.4 Hz, 2H), 3.90 (dt, *J* = 10.8, 5.5 Hz, 2H), 3.72 (ddd, *J* = 10.0, 4.9, 2.5 Hz, 2H), 3.60 (ddd, *J* = 9.9, 7.8, 5.1 Hz, 2H), 2.40 (t, *J* = 6.9 Hz, 4H), 2.06 (s, 6H), 2.02 (s, 6H), 2.00 (s, 6H), 1.97 (s, 6H), 1.85–1.75 (m, 4H); ^{13}C NMR (101 MHz, CD_2Cl_2) δ 171.0, 170.5, 169.9, 169.8, 101.5, 80.4, 73.2, 72.4, 71.8, 69.0, 68.7, 66.2, 62.5, 61.8, 60.9, 28.5, 21.1, 21.0, 21.0, 21.0, 16.5; HRMS (m/z): $[M]^+$ calcd. for $C_{42}H_{50}O_{20}$, 874.28594; found 874.28813.

Synthesis of docosa-4,6,8,10,12,14,16,18-octayne-1,22-diyl bis(2,3,4,6-tetra-*O*-acetyl-β-D-glucopyranoside) Glu[8]. In a flask shielded from light with aluminum foil, 11'-triisopropyl-silylundeca-4',6',8',10'-tetraynyl 2,3,4,6-tetra-*O*-acetyl-β-D-glucopyranoside (370.0 mg, 0.62 mmol) was dissolved in MeCN (2 mL) and DCM (2 mL). Silver fluoride (90.0 mg, 0.709 mmol) and copper(II) bromide (30.0 mg, 0.134 mmol) were consecutively added, and the

resulting mixture was stirred for 48 h. The mixture was purified by column chromatography (silica gel, DCM/acetone 20:1) to yield **Glu[8]** as a yellow solution. For analytical purposes, $CDCl_3$ (10 mL) was added, and the mixture was concentrated *in vacuo*.

^{1}H NMR (400 MHz, $CDCl_3$) δ 5.19 (dd, *J* = 10.2, 8.8 Hz, 2H, *H3*), 5.05 (t, *J* = 9.8 Hz, 2H, *H4*), 4.95 (dd, *J* = 9.7, 8.0 Hz, 2H, *H2*), 4.47 (d, *J* = 8.2 Hz, 2H, *H1*), 4.24 (dd, *J* = 12.3, 4.7 Hz, 2H, *H6*), 4.12 (dd, *J* = 12.2, 2.4 Hz, 2H, H6), 3.92 (dt, *J* = 10.1, 5.2 Hz, 2H, OC*H*H), 3.68 (ddd, *J* = 9.8, 4.7, 2.3 Hz, 2H, *H5*), 3.58 (ddd, *J* = 9.8, 8.0, 4.7 Hz, 2H, OC*H*H), 2.41 (t, *J* = 6.8 Hz, 4H, CCCH_2), 2.07 (s, 6H, CH_3), 2.03 (s, 6H, CH_3), 2.01 (s, 6H, CH_3), 1.99 (s, 6H, CH_3), 1.89–1.72 (m, 4H); ^{13}C NMR (101 MHz, $CDCl_3$) *δ* 170.8, 170.3, 169.5, 169.4, 101.0, 81.3, 72.8, 72.0, 71.4, 68.5, 68.0, 66.2, 63.4, 63.3, 62.9, 62.3, 62.0, 61.6, 60.6, 27.8, 20.8, 20.8, 20.7, 20.7, 16.1; HRMS (m/z): $[M]^+$ calcd. for $C_{50}H_{50}O_{20}$, 970.28954; found, 970.28938.

Synthesis of hexacosa-4,6,8,10,12,14,16,18,20,22-decayne-1,26-diyl bis(2,3,4,6-tetra-*O*-acetyl-β-D-glucopyranoside) Glu[10]. In a flask shielded from light with aluminum foil, 13'-tri-isopropylsilyltrideca-4',6',8',10',12'-pentaynyl 2,3,4,6-tetra-*O*-acetyl-β-D-glucopyranoside (205.0 mg, 0.307 mmol) was dissolved in MeCN (2 mL) and DCM (2 mL). Silver fluoride (45.0 mg, 0.355 mmol) and copper(II) bromide (90.0 mg, 0.403 mmol) were consecutively added, and the resulting mixture was stirred for 48 h. The mixture was purified by column chromatography (silica gel, DCM/acetone 9:1) to yield **Glu[10]** as a yellow solution. For analytical purposes, $CDCl_3$ (10 mL) was added, and the mixture was concentrated *in vacuo*.

^{1}H NMR (400 MHz, $CDCl_3$) *δ* 5.20 (t, *J* = 9.5 Hz, 2H), 5.07 (t, *J* = 9.7 Hz, 2H), 4.97 (dd, *J* = 9.7, 8.0 Hz, 2H), 4.48 (d, *J* = 8.0 Hz, 2H), 4.25 (dd, *J* = 12.3, 4.8 Hz, 2H), 4.13 (dd, *J* = 12.3, 2.4 Hz, 2H), 3.93 (dt, *J* = 10.2, 5.4 Hz, 2H), 3.69 (ddd, *J* = 10.1, 4.7, 2.4 Hz, 2H), 3.59 (ddd, *J* = 9.7, 8.1, 4.7 Hz, 2H), 2.42 (t, *J* = 6.8 Hz, 4H), 2.08 (s, 6H), 2.04 (s, 6H), 2.02 (s, 6H), 2.00 (s, 6H), 1.91–1.73 (m, 4H); ^{13}C NMR (101 MHz, $CDCl_3$) *δ* 170.8, 170.4, 169.6, 169.5, 101.0, 81.5, 72.8, 72.0, 71.4, 68.5, 68.0, 66.2, 63.8, 63.6, 63.5, 63.1, 62.6, 62.1, 62.0, 61.5, 60.6, 27.8, 20.9, 20.8, 20.8, 20.8, 16.2; HRMS (m/z): $[M]^+$ calcd. for $C_{54}H_{50}O_{20}$, 1018.28954; found, 1018.28794.

Synthesis of triaconta-4,6,8,10,12,14,16,18,20,22,24,26-dodecayne-1,30-diyl bis(2,3,4,6-tetra-*O*-acetyl-β-D-glucopyranoside) Glu[12]. In a flask shielded from light with aluminum foil, 15'-triisopropylsilylpentadeca-4',6',8',10',12',14'-hexaynyl 2,3,4,6-tetra-*O*-acetyl-β-D-glucopyranoside (185.2 mg, 0.268 mmol) was dissolved in THF (50 mL) and water (10 mL). Cesium fluoride (52.9 mg, 0.348 mmol) was added, and the resulting mixture was stirred for 2 h. The mixture was diluted with ethyl acetate (50 mL) and *n*-pentane (50 mL), washed three times with saturated aqueous NH_4Cl solution, and once with saturated aqueous NaCl solution. The organic phase was dried over Na_2SO_4, concentrated *in vacuo* to a volume of ca. 10 mL, and purified by column chromatography (silica gel, heptane/EtOAc 1:1, R_f: 0.33). The combined fractions of the pentadeca-4',6',8',10',12',14'-hexynyl 2,3,4,6-tetra-*O*-acetyl-β-D-glucopyranoside were concentrated *in vacuo* to a volume of ca. 20 mL, DCM (30 mL), $Cu(OAc)_2 \cdot H_2O$ (267.5 mg, 1.34 mmol), and 2,6-dimethylpyridine (0.93 mL, 8.04 mmol) were added, and the resulting mixture was stirred for 48 h. To this mixture 1 M HCl (100 mL) was added and the aqueous layer was extracted with DCM. The combined organic phases were washed with saturated NaCl solution, dried over Na_2SO_4, and concentrated *in vacuo*. Column chromatography (silica gel; EtOAc/*n*-pentane 4:1) yielded **Glu[12]** as a yellow solution. For analytical purposes, $CDCl_3$ (10 mL) was added, and the mixture was concentrated *in vacuo*.

TLC (EtOAc:*n*-pentane 80:20 v/v): R_f = 0.61; ^{1}H NMR (500 MHz, $CDCl_3$) δ 5.21 (t, J = 9.5 Hz, 2H), 5.08 (t, J = 9.7 Hz, 2H), 4.97 (dd, J = 9.7, 8.0 Hz, 2H), 4.49 (d, J = 8.0 Hz, 2H), 4.26 (dd, J = 12.3, 4.8 Hz, 2H, *H6*), 4.13 (dd, J = 12.3, 2.4 Hz, 2H, *H6*), 3.94 (dt, J = 10.5, 5.3 Hz, 2H), 3.70 (ddd, J = 10.0, 4.8, 2.4 Hz, 2H), 3.59 (ddd, J = 9.6, 8.2, 4.7 Hz, 2H), 2.43 (t, J = 6.5 Hz, 4H), 2.05 (s, 6H), 2.05 (s, 6H), 2.03 (s, 6H), 2.01 (s, 6H), 1.91–1.73 (m, 4H). ^{13}C NMR (126 MHz, $CDCl_3$) δ 170.8, 170.4, 169.6, 169.5, 101.1, 81.6, 72.8, 72.0, 71.4, 68.5, 68.0, 66.2, 64.0, 63.8, 63.7, 63.5, 63.2, 62.9, 62.5, 62.0, 61.4, 60.5, 60.5, 27.8, 20.9, 20.8, 20.8, 20.8, 16.2; HRMS (m/z): $[M]^+$ calcd. for $C_{58}H_{50}O_{20}$, 1066.28954; found, 1066.28776.

5. Supplementary Method 3

^{1}H NMR spectrum (400 MHz, CD_2Cl_2) of **Glu[4]**.

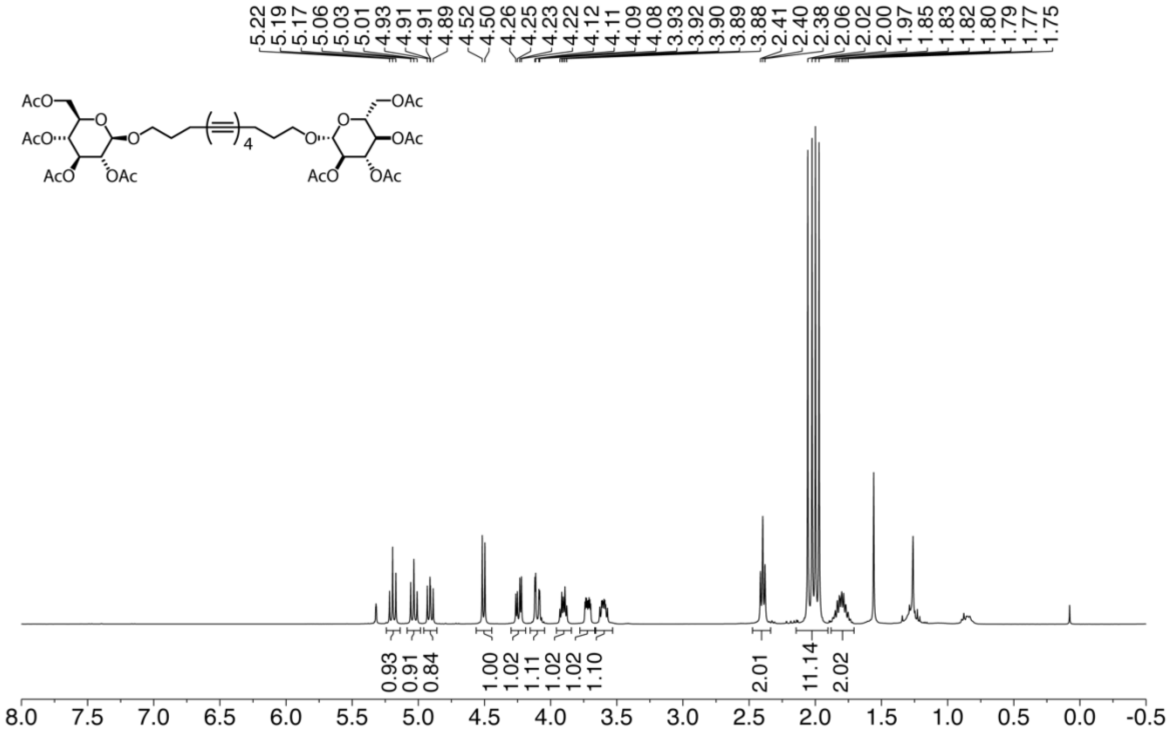

^{13}C NMR spectrum (101 MHz, CD_2Cl_2) of **Glu[4]**.

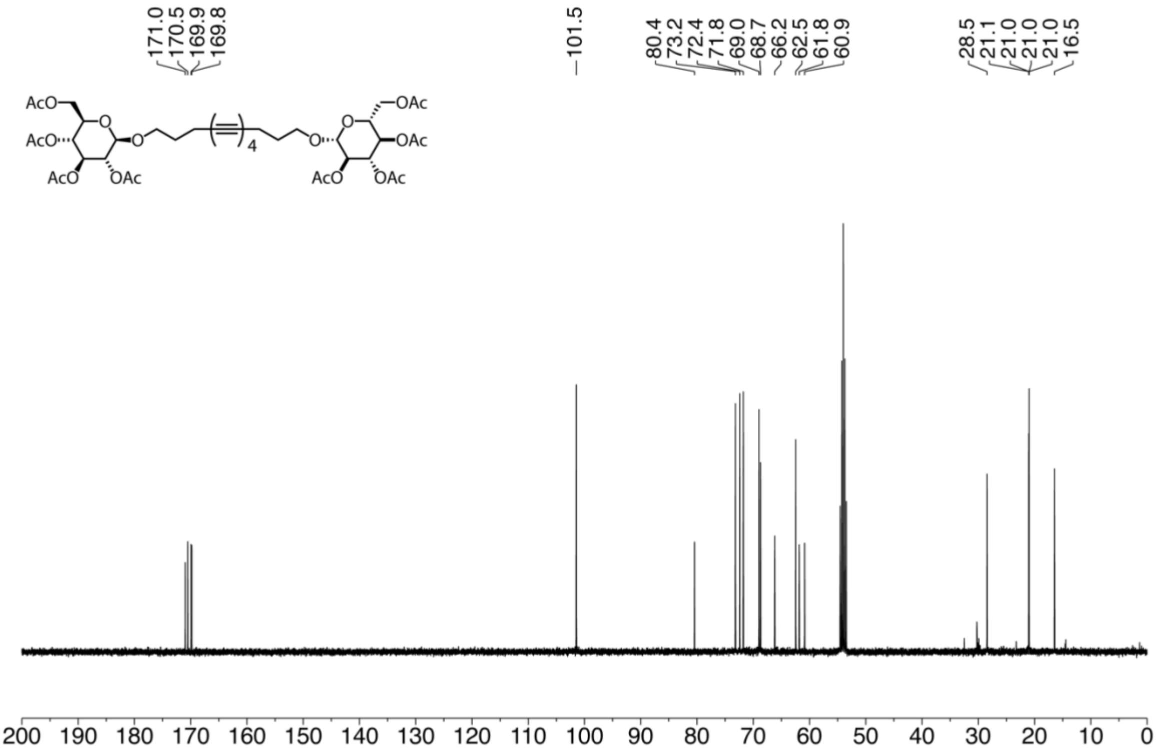

1H NMR spectrum (400 MHz, $CDCl_3$) of **Glu[8]**.

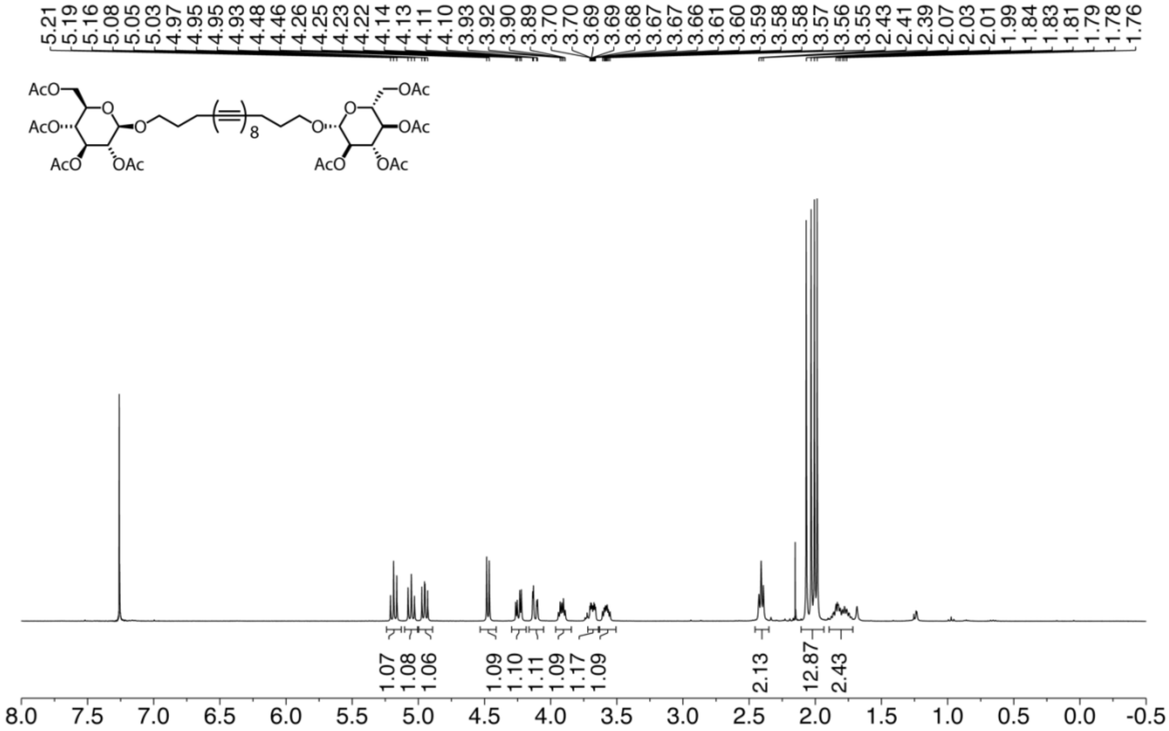

13C NMR spectrum (101 MHz, $CDCl_3$) of **Glu[8]**.

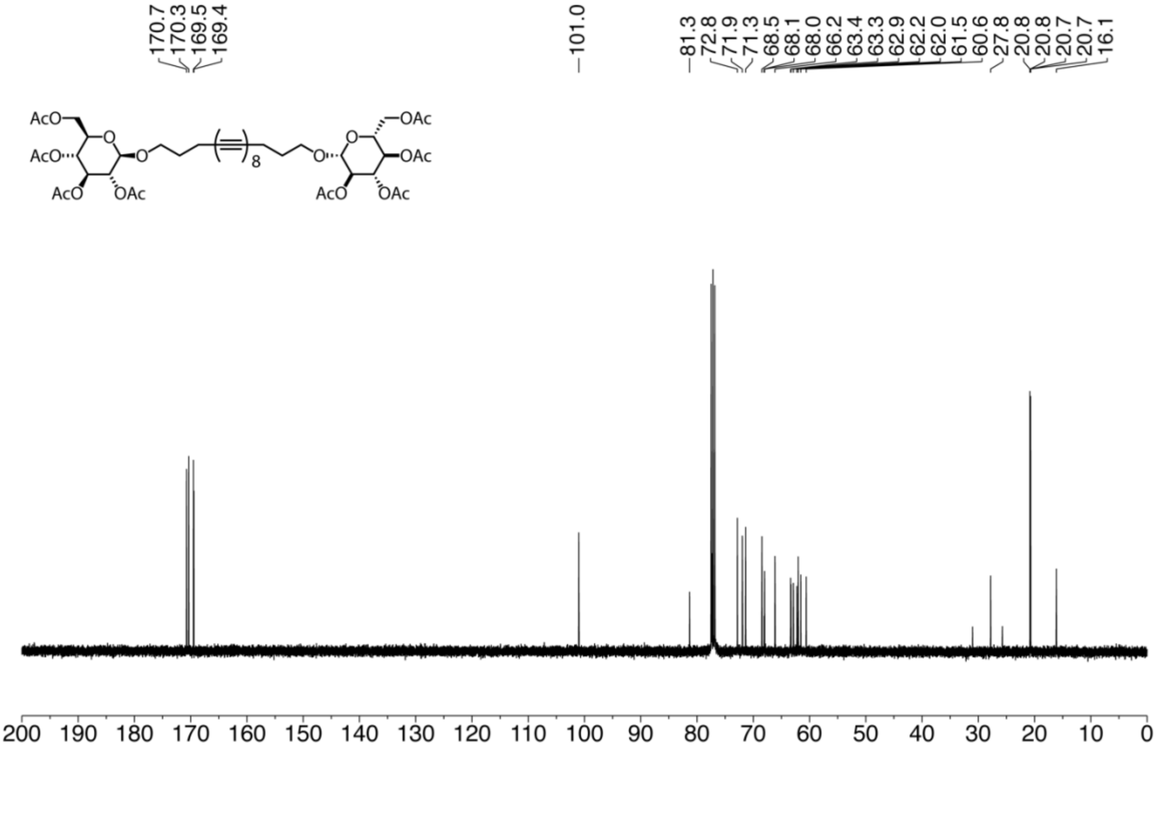

^{1}H NMR spectrum (400 MHz, $CDCl_3$) of **Glu[10]**.

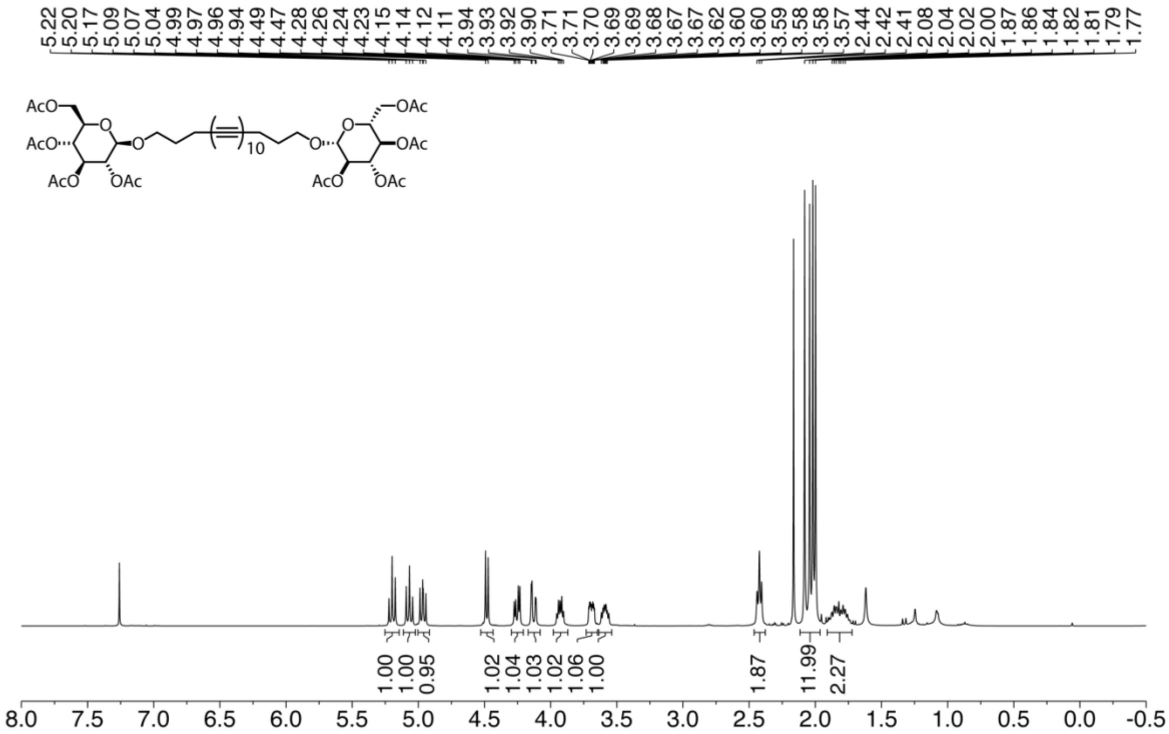

^{13}C NMR spectrum (101 MHz, $CDCl_3$) of **Glu[10]**.

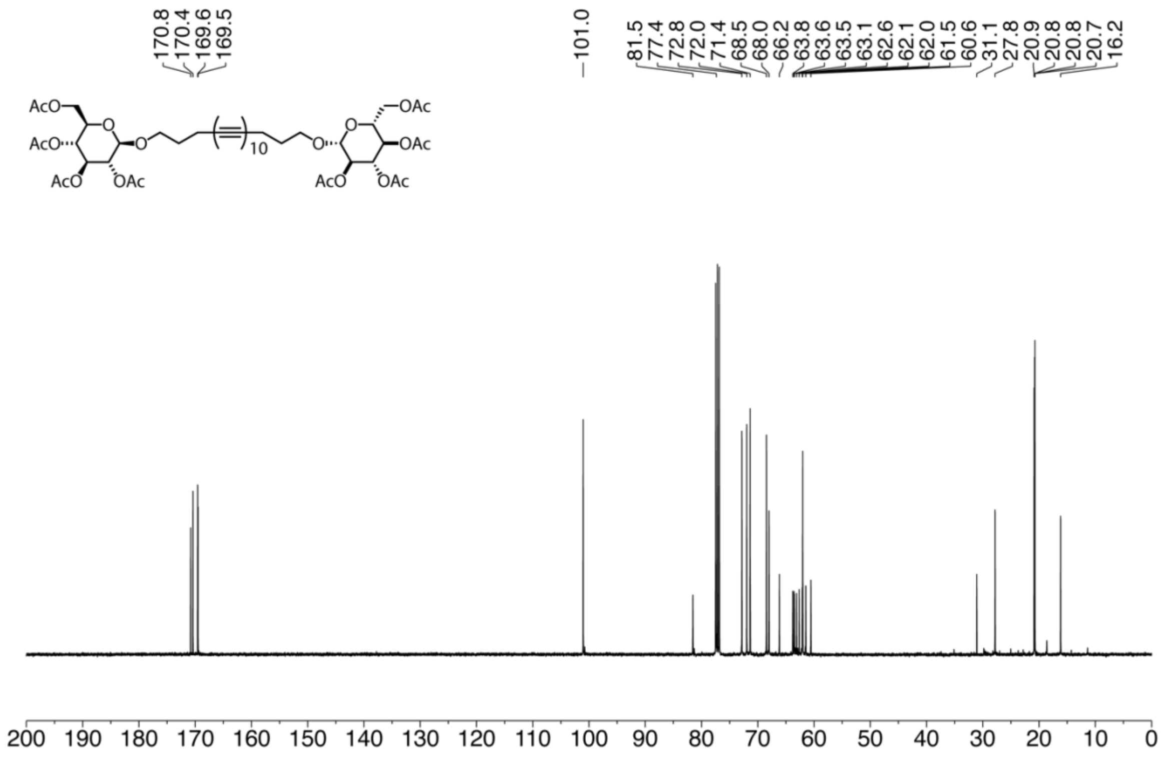

^{1}H NMR spectrum (500 MHz, $CDCl_3$) of **Glu[12]**.

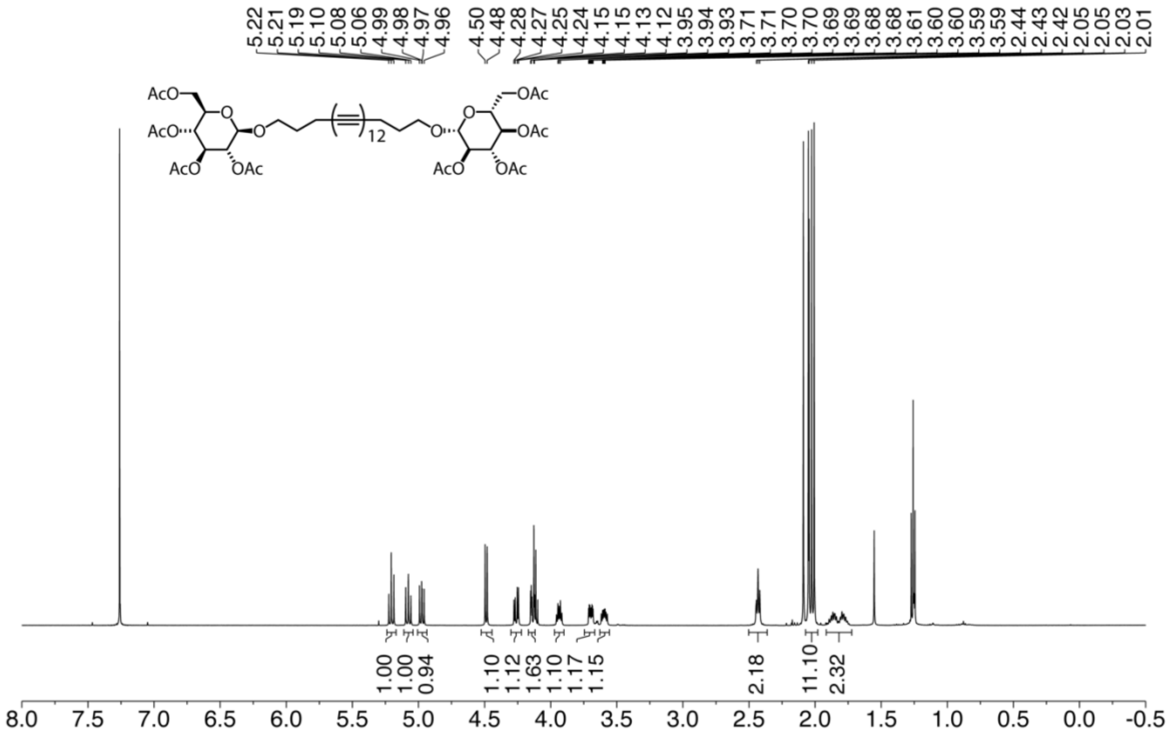

^{13}C NMR spectrum (126 MHz, $CDCl_3$) of **Glu[12]**.

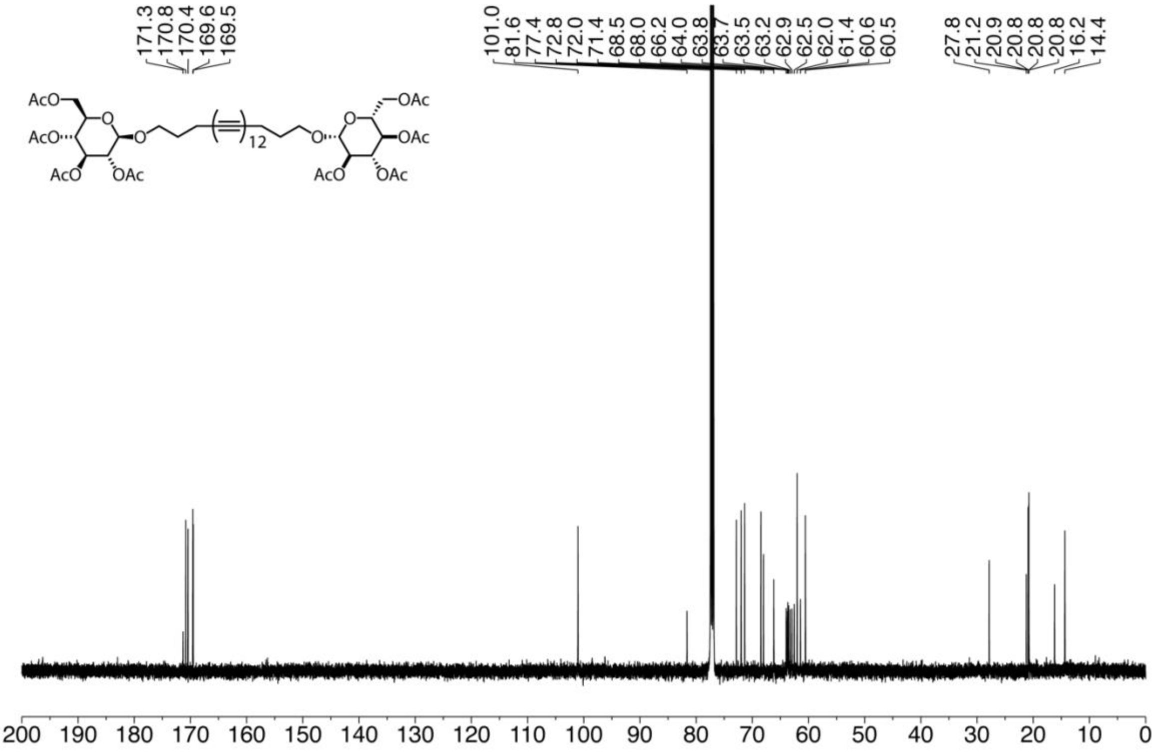

6. Supplementary References

1. Chalifoux, W. A. & Tykwinski, R. R. Synthesis of polyynes to model the sp-carbon allotrope carbyne. Nat. Chem. 2, 967–971 (2010).
2. Mardelli, M. & Olmsted, J., III. Calorimetric determination of the 9, 10-diphenyl-anthracene fluorescence quantum yield. Journal of Photochemistry 7, 277–285 (1977).
3. Snellenburg, J. J., Laptenok, S. P., Seger, R., Mullen, K. M. & Stokkum, I. H. M. V. Glotaran: A Java-Based Graphical User Interface for the RPackage TIMP. J. Stat. Softw. 49, (2012).
4. van Stokkum, I. H. M., Larsen, D. S. & van Grondelle, R. Global and target analysis of time-resolved spectra. Biochim. Biophys. Acta Bioenerg. 1657, 82–104 (2004).
5. Mullen, K. M. & Stokkum, I. H. M. V. TIMP: An RPackage for Modeling Multi-Way Spectroscopic Measurements. J. Stat. Softw. 18, (2007).
6. Schrettl, S. et al. Facile synthesis of oligoyne amphiphiles and their rotaxanes. Chem. Sci. 6, 564–574 (2015).
